# Chronic Bee Paralysis Virus and *Nosema ceranae* Experimental Co-Infection of Winter Honey Bee Workers (*Apis mellifera L*.) 

**DOI:** 10.3390/v5092282

**Published:** 2013-09-19

**Authors:** Ivan Toplak, Urška Jamnikar Ciglenečki, Katherine Aronstein, Aleš Gregorc

**Affiliations:** 1 Veterinary Faculty, University of Ljubljana, Gerbičeva 60, 1000 Ljubljana, Slovenia; E-Mails: urska.jamnikar@vf.uni-lj.si; 2 Honey Bee Breeding, Genetics and Physiology Lab, USDA-ARS, 1157 Ben Hur Road, Baton Rouge, LA 70820, USA; E-Mail: kate.aronstein@ars.usda.gov; 3 Agricultural Institute of Slovenia, Hacquetova 17, 1001 Ljubljana, Slovenia; E-Mail: ales.gregorc@kis.si; 4 Faculty of Agriculture and Life Sciences, University of Maribor, Pivola 10, 2310 Hoče, Slovenia

**Keywords:** *Apis mellifera*, experimental infection, Chronic Bee Paralysis Virus (CBPV), *Nosema ceranae*, interaction

## Abstract

Chronic bee paralysis virus (CBPV) is an important viral disease of adult bees which induces significant losses in honey bee colonies. Despite comprehensive research, only limited data is available from experimental infection for this virus. In the present study winter worker bees were experimentally infected in three different experiments. Bees were first inoculated per os (*p/o*) or per cuticle (*p/c*) with CBPV field strain M92/2010 in order to evaluate the virus replication in individual bees. In addition, potential synergistic effects of co-infection with CBPV and *Nosema ceranae* (*N. ceranae*) on bees were investigated. In total 558 individual bees were inoculated in small cages and data were analyzed using quantitative real time RT-PCR (RT-qPCR). Our results revealed successful replication of CBPV after *p/o* inoculation, while it was less effective when bees were inoculated *p/c*. Dead bees harbored about 1,000 times higher copy numbers of the virus than live bees. Co-infection of workers with CBPV and *N. ceranae* using either method of virus inoculation (*p/c or p/o*) showed increased replication ability for CBPV. In the third experiment the effect of inoculation on bee mortality was evaluated. The highest level of bee mortality was observed in a group of bees inoculated with CBPV *p/o*, followed by a group of workers simultaneously inoculated with CBPV and *N. ceranae p/o*, followed by the group inoculated with CBPV *p/c* and the group with only *N. ceranae p/o.* The experimental infection with CBPV showed important differences after *p/o* or *p/c* inoculation in winter bees, while simultaneous infection with CBPV and *N. ceranae* suggesting a synergistic effect after inoculation.

## 1. Introduction

The honeybee, *Apis mellifera* L., is a well-known honey producer and plays a major role in agriculture by assisting in the pollination of a wide variety of crops. In recent years, honeybees have been strangely disappearing from their hives and strong colonies have become weak and died [1,2]. Viral infections are the least understood of honeybee diseases, due to the lack of information on the mechanisms underlying potential disease outbreaks and limited experimental data available on their different modes of spread, transmission, and persistence [3]. Honeybee colony losses have occurred in Europe and America that cannot be attributed to the Varroa mite (*Varroa destructor*), so an unknown combination of stressors is suspected to be the cause, including other pathogens [1,2,4]. Thus knowledge of the spreading mechanism and synergistic effect of different pathogens within the hives is crucial for understanding bee disease dynamics [4,5,6]. 

Chronic bee paralysis virus (CBPV) causes a contagious disease of adult honey bees that manifests as chronic paralysis syndrome leading to death of infected bees [7,8]. The morphology of the CBPV particles and the multipartite organization of the RNA genome are unique among other bee viruses, as most of them are picorna-like viruses in the *Dicistroviridae* and *Iflaviridae* family with symmetric particles and monopartite-positive single-stranded RNA genomes. CBPV is currently classified as a positive single-stranded RNA virus but it has not been assigned to any family or genus yet. The CBPV was first isolated in 1963 and infection may negatively affect any colony in the apiary, weak or strong, resulting in thousands of dead individuals in front of the hives [7,8]. Two forms of the disease are currently well defined. The first form of the disease manifests as abnormal trembling of the body and wings. Symptomatic bees are not able to fly, often crawl on the ground and eventually die in front of the colony. They may also have bloated abdomens due to distension of the honey sac and huddle together on top of the bee cluster. The second form of the disease is manifested by the hairless‑black syndrome [9,10]. Bees inoculated in the laboratory show clinical symptoms of the disease 5–6 days post inoculation (dpi), similar to symptoms observed in naturally infected bees [7,11]. CBPV can also persist asymptomatically [12,13].

CBPV is found in bees worldwide; the most likely result of intensive commercial exchange of bees and hive materials [8]. In France, the prevalence of CBPV is 28% [14], while in some other European countries the prevalence in winter or summer bees is much lower (e.g., 9% in Austria, 4% in Denmark, 0% in Hungary and 15%–18.3% in Slovenia) [15,16,17,18,19].

Pathogen–pathogen interaction are known for *Varroa destructor* associated with different viruses and pathogen together may have a significant impact on health and longevity of the individual honey bees as well as on the overall colony survival and productivity [6,20,21]. Synergistic effect between microsporidia fungal pathogen *Nosema apis* and several honey bee viruses such as filamentous virus (FV), bee virus Y (BVY) and black queen cell virus (BQCV) were reported years ago [22], while no association was found between *Nosema ceranae* (*N. ceranae*) and deformed wing virus (DWV) [4,23]. The infection of the midgut epithelium occurs per os when the honey bee ingests food contaminated with *N. ceranae* spores [24]. The reproduction life cycle of *N. ceranae* is completed in less than three days in Asian bees [25]. However, pathology of the disease caused by *N. ceranae* is less known in the western bee (*Apis mellifera*). Extensive lysis of epithelial cells induced by *N. apis* showed massive cell death and starvation of bees due to impaired nutrient absorption [26]. Nosema spores can be detected using light phase contrast microscopy or DNA amplification [27,28]. Bees with evident “dysentery” are normally associated with *N. apis* pathology and can also spread CBPV via feces [29]. Because CBPV was frequently detected in Slovenia [18,19] and limited experimental data is available for CBPV field samples were of great interest in order to establish some additional characteristics of the CBPV field strain and synergistic effects between CBPV and *N. ceranae*.

The present study describes the results of three experimental infection of individual winter worker bees with CBPV (field strain M92/2010) and co-infection with CBPV and *N. ceranae* spores. The main objectives of this study are: (1) to provide the relevant data of CBPV replication in winter bees as a potential cause of individual winter bee mortality and (2) to evaluate the synergistic effects of two pathogens in caged bees. The viral loads in individual bees were detected by RT-qPCR in CBPV infected bees or in bees co-infected with CBPV and *N. ceranae*. 

## 2. Results and Discussion

**Figure 1:**
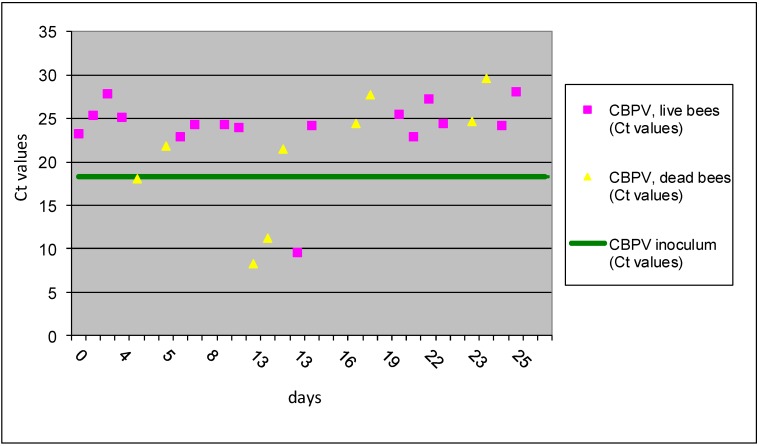
The results of Ct values obtained with RT-qPCR for group I A are presented for individual bees inoculated CBPV *p/c*. Horizontal line indicates the average Ct value of 18.35 for the CBPV inoculums.

**Figure 2:**
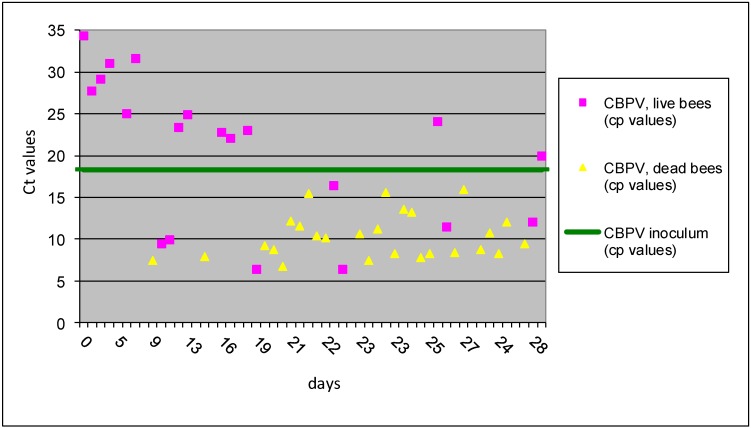
The results of Ct values obtained with RT-qPCR for I B group are presented for individual bees inoculated CBPV *p/o*. Horizontal line indicates the average Ct value of 18.35 for the CBPV inoculums.

The results of this study revealed successful replication of CBPV in experimentally infected bees after *p/o* inoculation, while the replication of CBPV was less effective when bees were inoculated *p/c* (Table 1, Figure 1 and Figure 2)*.* Inoculated bees with CBPV *p/o*, harbored about 1,000 times higher copy numbers of the virus in dead bees than live bees. This is consistent with some previous observations, that high viral load can be important indicator for bee mortality [6,11]. Co-infection of workers with CBPV and *N. ceranae* showed increased replication ability for CBPV when using *p/c* inoculation, suggesting synergistic effect of *N. ceranae* on CBPV replication. The highest level of bee mortality was observed in group of bees inoculated with CBPV *p/o*, confirming that this virus is an important pathogen for adult bee. In group of workers simultaneously inoculated with CBPV and *N. ceranae p/o* we observed the same rate of mortality than in group inoculated with CBPV *p/c*, followed by the group inoculated with only *N. ceranae p/o.*

**Table 1:** The scheduling of the chronic bee paralysis virus (CBPV) and *N. ceranae* spores inoculations in three experiments performed in this study. Winter workers were caged in each group and individually treated as indicated in last column. CBPV indicates chronic bee paralysis virus inoculum (field strain M92/2010), *N. ceranae* indicates *Nosema ceranae* spores application, and acronym RPMI indicates RPMI-1640 medium, *p/c* indicates *per cutis* treatment applied on to the intersegmental membrane between the second and third abdominal tergite; *p/o* indicates *per os* treatment.

Experiment No	Cages (Group)	Treatment			
Experiment I	I A	CBPV	*p/c*	-	
	I B	CBPV	*p/o*	-	
	I C (control)	RPMI	*p/c*	-	
	I D (control)	RPMI	*p/o*	-	
Experiment II	II A	CBPV	*p/c*	*N. ceranae*	*p/o*
	II B	CBPV	*p/o*	*N. ceranae*	*p/o*
	II C	-		*N. ceranae*	*p/o*
	II D (control)	RPMI	*p/o*	-	
Experiment III	III A	-		*N. ceranae*	*p/o*
	III B	CBPV	*p/o*	*N. ceranae*	*p/o*
	III C	CBPV	*p/c*	-	
	III D	CBPV	*p/o*	-	
	III E (control)	RPMI	*p/o*	-	

### 2.1 Experiment No I: Workers Inoculated with CBPV “per cutis” (p/c) or “per os” (p/o)

By testing different treatment groups, we confirmed that CBPV field strain was infectious and was replicated more efficiently when bees were infected *p/o* in comparison to *p/c* inoculation method (Figure 1 and Figure 2). This is a very important observation that can explain the significance of *p/o* infection during transmission of CBPV between bees. The differences between *p/o* and *p/c* inoculation can be evaluated only in experimentally infections and in this study was proved for the first time the different possibility of outcome. These types of horizontal transmission are possible both, within beehives and between apiaries. The inoculation “per cuticle” is simulating the infection “per contact” and the results of this study showed successful replication of virus in 12% of inoculated bees, which is low percent, but could be important way for the CBPV spreading within colony and between apiaries. The obtained Ct values for individual bees of group I A by RT-qPCR method revealed that three workers collected between 9 and 13 dpi were detected with Ct values 8.98, 10.12 and 11.49, which represent about 1,000-times higher copy number than of the CBPV RNA detected in inoculum (Figure 1). In the remaining 22 workers collected from the group I A no replication of CBPV was observed after *p/c* inoculation. 

In group I B (CBPV *p/o* inoculation), 20 live bees (43%) collected during 28 dpi had an average Ct 20.54 ± 8.7. In contrast, 26 dead bees (57%) collected from the same treatment group had the average Ct values 10.34 ± 2.7. These results showed significantly higher levels of viral RNA copies (>1,000 times) in dead bees vs. live bees, demonstrating the positive correlation between CBPV viral load and bee mortality, similar as was described previously [11]. The first dead bees with high copy number of CBPV were detected at day 7 dpi, but the majority of dead bees with the elevated CBPV copy numbers were detected after 19 dpi (Figure 2)*.* However, experiment No I was not focused directly on mortality, because the sampling procedure was directed towards the detection of virus replication in individual bees, through 28 days after inoculation. 

As expected, all bees in the two control groups (group I C and I D) were found CBPV negative by RT-qPCR (data not shown). 

### 2.2. Experiment No II: CBPV Co-Infection with N. ceranae

**Figure 3:**
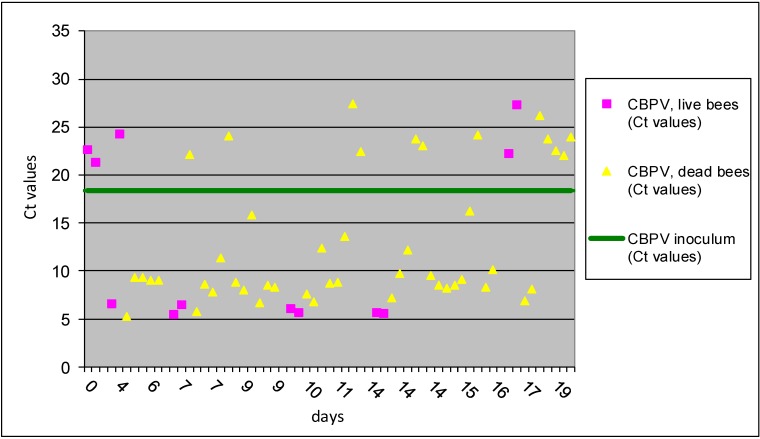
The results of Ct values obtained with RT-qPCR for II A group are presented for individual bees inoculated with CBPV *p/c* and co-infection with *N. ceranae p/o*. Horizontal line indicates the average Ct value (18.35) of the CBPV inoculum.

In experiment No II, the two modes of CBPV viral infection (*p/o* vs. *p/c*) were tested in conjunction with *N. ceranae p/o* co-infection. Numbers of detected spores in live and dead bees were counted and presented for each group (Table 2). We have found that *p/c* inoculation with CBPV alone (group I A) triggered viral replication only in 12% of infected bees, while 70,6% of bees were successfully infected with the much higher incidence of virus replications in bees co-infected with CBPV *p/c* and *Nosema* spores *p/o*. CBPV can be an important pathogen which replication is trigger with *N. ceranae* and together may be responsible for weakling honeybee colony. This is supported also with the results in experiment No II, showing significant higher average number of *N. ceranae* spores in dead bees, comparing to detected number of spores in live bees (Table 2). These data support that *N. ceranae* can be important pathogen in co-infection with CBPV not only alone, as was reported in previous observations [21]. Twelve live bees (20.6%) co-infected with CBPV *p/c* and *N. ceranae p/o* (group II A) had an average virus Ct value of 13.27 ± 9.2 and 46 dead bees (79.4%) had an average Ct value of 12.99 ± 6.7, confirming successful replication of CBPV in majority of bees from this group. The first bees in II A group with evidence of virus replications were sampled on day 3 dpi (Figure 3), which is much earlier than observed in I A group, 9 dpi (Figure 1). Data analysis showed that 70.6% of individual bees in group II A treatment had the average level of CBPV which is about 1,000-times higher than CBPV copy number in the inoculum. 

**Table 2:** Results for five groups of caged bees are presented for experiments II and III, which were inoculated with *N. ceranae* spores. Only average number of spores is presented for each group, after number of spores was counted in individual bees by adding 1 mL of RPMI per bee, using Bürker haemocytometer [27]. CBPV indicates chronic bee paralysis virus inoculum (field strain M92/2010), *N. ceranae* indicates *Nosema ceranae* spores application, and acronym RPMI indicates RPMI-1640 medium, *p/c* indicates *per cutis* treatment applied on to the intersegmental membrane between the second and third abdominal tergite; *p/o* indicates *per os* treatment.

Experiment No	Cages (Group)	Treatment		Average number of detected *N. ceranae* spores; counts ×10^6^ spores/bee	
		CBPV	*N. ceranae*	Live bees	Dead bees
Experiment II	II A	*p/c*	*p/o*	23.55	33.3
	II B	*p/o*	*p/o*	43.3	106.1
	II C	-	*p/o*	57.4	103.5
	II D	RPMI	-	0	0
Experiment III	III A	-	*p/o*	-	93.4
	III B	*p/o*	*p/o*	-	85.3
	III C	*p/c*	-	-	0
	III D	*p/o*	-	-	0
	III E	RPMI	-	-	0

**Figure 4:**
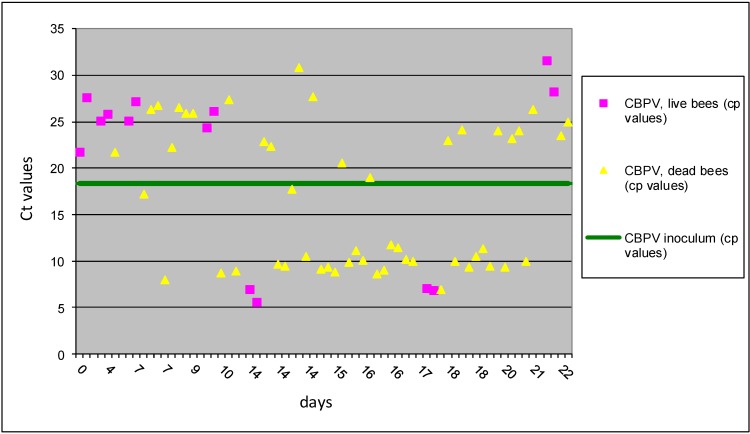
The results of Ct values obtained with RT-qPCR for II B group are presented for individual bees inoculated with CBPV *p/o* and *N. ceranea p/o*. Horizontal line indicates the average Ct value (18.35) of the CBPV inoculum.

Results of CBPV and *Nosema* co-infected group, *p/o* (II B group) showed that half of 64 bees had Ct values above the level of inoculum and 32 bees from this group had Ct value below of inoculum (Figure 4). The increased number of bees with virus replication was observed after 10 dpi, and this observation is recorded also in I B group from this study. But the obtained results of Ct values showed an important difference with much higher variability of Ct values in the II B group. However, higher bee mortality was observed in group II B, where combined co-infection with the oral rout of inoculation was performed. Our results support previous observation indicating that the honey bee digestive system could be the primary route of CBPV infection [29]. *N. ceranae* causes damage to the mid-gut epithelial cells and actively suppress honeybee immune response, which could increase the virulence of viral pathogens. In addition, our results showed that co-infection with CBPV and *N. ceranae* can increase virus replication also after CBPV *p/c* and *Nosema* spores *p/o* inoculation, but the mechanisem of this is not known. Nevertheless, this observation can suggest important synergistic effect between both pathogens. 

All bees in control groups II C and II D were CBPV negative by RT-qPCR (data not shown). 

### 2.3. Experiment No III: The Mortality Test

**Figure 5:**
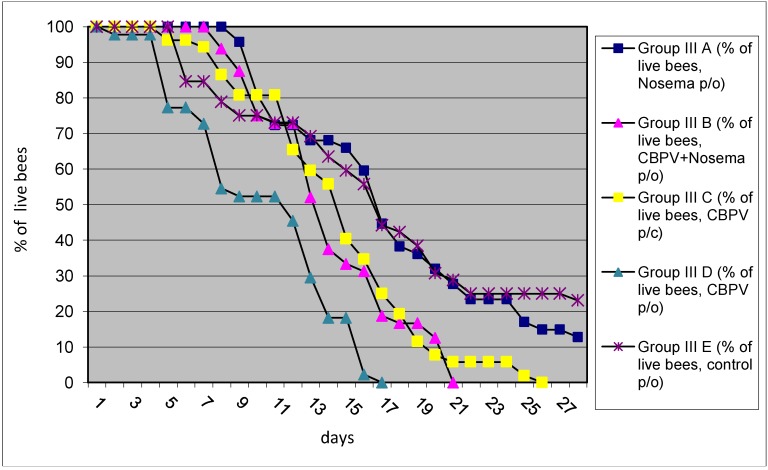
The percentage of live bees remaining after each day is presented for the five groups (the mortality test). In each group, bees were individually inoculated as follows: group III A, *N. ceranae* spores *p/o*; group III B, CBPV and *N. ceranae* spores *p/o*; group III C, CBPV *p/c*; group III D, CBPV *p/o*; group III E, control group. Only dead bees were sampled daily.

The highest bee mortality was observed in group III D (workers inoculated with CBPV *p/o*), followed with group III B (workers simultaneously inoculated with CBPV and *N. ceranae p/o*), group III C (inoculated workers with CBPV *p/c*) and finally with group III A (workers received only *N. ceranae* spores), the lowest bee mortality was found in control group III E (Figure 5). Inoculation of bees with the field strain CBPV 92/2010 and/or *N. ceranae* revealed the negative impact on winter bee survival. The data obtained during the experiment confirmed higher mortality in winter bees in comparison to control groups and sporadic clinical cases of CBPV infection are probably associated also with this pathogen. These data support our previous observation, that CBPV infection can be one of the most important viruses resulting in number of colony losses [18,19]. 

Sequence analysis of field strain CBPV 92/2010 showed 99.6% nucleotide and 100% amino acids identity with isolates BE 78, H4 300, R1-C4, R2-C102, L-4, BE-104 detected in France (GenBank accession numbers FJ345308, FJ345317, FJ345322, FJ345310, FJ345306). These data suggest that similar strains CBPV are present also in France. The comparison of 540 nt long sequences obtained from inoculated groups I A, I B, II A, II B, III B, III C and III D revealed 100% nucleotide identity to each other. This data confirmed, that only inoculated strain CBPV 92/2010 was detected from all experimental groups. 

### 2.4. Do the CBPV and N. ceranae Act Synergistically?

Our study confirmed that honey bee responses after experimental infection is complex with a number of factors affecting the replication of CBPV in individual bees and this can be even more complex on the bee colony levels. Although conditions of this study were not optimal due to a low level of other pathogens found in the inoculum (e.g., BQCV) and original experimental bee colonies (e.g., BQCV and DWV), we believe that the presence of BQCV had a minor impact on the results in adult bees in this study. Recently it was demonstrated that the life span of winter bees infected with DWV and *Varroa destructor* is reduced significantly [6]. Although bees used in our study were free of *Varroa destructor* the original colony was positive RT-PCR and the contribution of DWV on the mortality of caged bees cannot be excluded. This is supported by the fact that the mortality of winter bees was observed also in control groups, although lower than in inoculated group with CBPV (Figure 5). Furthermore, the multiple virus infections are frequently detected in worker bees, thus the complex of co-infections or multiple infections is usually present in honeybee colonies. In Slovenia, about 80%–90% of adult bees were found BQCV positive and 97% of samples contain at least one bee virus, thus virus free bees was difficult to find for this study [19]. The two pathogens tested in this study, CBPV and *N. ceranae,* act synergistically on CBPV replication especially when using *p/c* inoculation. The interpretation of the monitoring results from pool of bee samples with Ct values or viral load expression is rather average copy number, while the results in this study reflect some key points of the complexity of *in vivo* experimental infection in individual bees. Results obtained in this study need to be further evaluated also using the “short-living” summer bees. CBPV is widespread viral infection and can be responsible for heavy losses in worker bees. In the absence of honeybee cell line, experimental infection is the only method to reproduce the virus infection in honey bees and the present study showed some complexity of different responses on individual bees.

## 3. Experimental Section

### 3.1. Winter Worker Bee (Apis mellifera carnica) Used in Experiments

Two honey bee experimental colonies, originated from the same apiary, headed by Carniolan queens (*Apis mellifera carnica*) were used in this study conducted in Slovenia during winter of 2011. Prior to the experiment, normal size healthy colonies were examined for clinical signs of bee diseases.

Winter bees were collected in January 2011 by brushing them off the combs of these two colonies. Pools of about 200 winter workers from each colony were tested for the presence of six honey bee viruses (ABPV, BQCV, CBPV, DWV, SBV and KBV) using the RT-PCR [19]. Bees were also examined for the presence of *Nosema* spores using microscopy [27]. Both colonies were positive for BQCV and DWV and negative for ABPV, CBPV, SBV and KBV viruses and *Nosema* spp. For cage experiments, bees were selected from the two experimental colonies and randomly set up in cages and incubated at 28 °C and 65 % relative humidity for the duration of the study. Bees were provided with 50 % sugar solution (1:1; w/w) for the duration of the experiment. 

### 3.2. CBPV (Chronic Bee Paralysis Virus, Strain M92/2010) Inoculums

The CBPV field strain M92/2010 originated from paralyzed bees collected in May 2010 in Slovenia showing clinical symptoms of viral infection. A pool of paralyzed workers (n = 50) from the same apiary was collected and stored at −70 °C until use. Paralyzed bees (n = 50) were homogenized in 50 mL of RPMI-1640 (Gibco, UK) using ULTRA-TURRAX^®^ DT-20 Dispersing Tubes (IKA, Germany) and a pooled sample was tested for the presence of six honey bee viruses using RT-PCR method [19]. Only two of six viruses, CBPV and BQCV, were detected positive by RT-PCR. The average CBPV cycle threshold (Ct) value of original inoculum (10 replicates) was established by using RT-qPCR method (described in Section 3.6). After homogenization, the suspension was centrifuged for 15 min at 2,500 rpm and supernatant was recovered. Supernatant was filtered first through a 0.45 µM and then through 0.22 µM pore size membrane filters (Sartorius, UK) and stored in 5 mL aliquots at −70 °C until required. 

### 3.3. N. ceranae Inoculums

Workers used as a source of *N. ceranae* spores were obtained from the colony maintained at the experimental apiary, Agricultural Institute of Ljubljana, Slovenia. Molecular characterization of the spore inoculum was performed using PCR [24] and *N. ceranae* spore load was determined by using light phase contrast microscopy. Briefly, mid-guts were dissected from the infected workers, macerated and spores were washed three times by centrifugation in Insect Ringer’s solution. The concentration of spores was determined by counting spores in a Bürker haemocytometer. Fresh spore inoculums (61,400 spores per bee) were prepared on a daily basis using fresh bees. 

### 3.4 Experiment Design

Three experiments were performed using caged winter workers. In each cage, about 50 bees were randomly collected from two Carniolan honey bee colonies. Bees in cages were individually inoculated using three different individual treatments, as described in 3.4.1–3.4.3. Two (live or dead) bees were sampled in experiment No I and experiment No II in intervals of 2–3 days. Experiment No III was performed to establish the mortality rate of inoculated workers, thus only dead workers were sampled daily. All workers were inoculated individually using a fresh inoculum for each experimental group and sterile filter pipette tips for individual bee. 

#### 3.4.1. Experiment No I: Workers Inoculated with CBPV “per cutis” (*p/c*) or “per os” (*p/o*)

Winter workers used for experiment No I were divided into four cages (groups I A, I B, I C, I D), each group assigned to a different treatment (Table 1). 25 workers of group I A were inoculated individually with 2 µL of CBPV inoculum per contact to the inter-segmental membrane between the second and third abdominal tergite using new sterile filter pipette tips for individual bee. The treatment is further indicated as *per cutis* (*p/c*) treatment. 46 workers of the second group (I B) were inoculated per os (*p/o)* with 2 µL of CBPV inoculum per bee using new sterile filter pipette tips for individual bee, 26 workers of group I C were inoculated individually with 2 µL of macerated CBPV free and *N. ceranae* free bees in RPMI-1640 medium (Gibco, UK) *per cutis* (*p/c*, negative control group) and 20 workers of group I D were inoculated with 2 µL macerated CBPV free and *N. ceranae* free bees in of RPMI-1640 medium (Gibco, UK) *per os* (*p/o*, negative control group). *p/c* inoculation simulates the potential transmission of highly contagious diseases via contact; whereas *p/o* rout simulates ingestion of virus-contaminated foods and/or bee-to-bee transmission via trophallaxis.

#### 3.4.2 Experiment No II: CBPV Co-Infection with *N. ceranae*

Winter workers were divided into four cages (groups) as follows: (group II A) workers (n = 58) were inoculated individually with 2 µL of CBPV *p/c* and 2 µL of *N. ceranae p/o*; (group II B) workers (n = 64) received individually 2 µL of CBPV and 2 µL of *N. ceranae*, both *p/o*; (group II C) workers (n = 62) were inoculated individually only with *N. ceranae p/o*; (group II D) workers (n = 22) were inoculated individually with 2 µL of macerated CBPV free and *N. ceranae* free bees in RPMI-1640 medium *p/o* (negative control group).

#### 3.4.3. Experiment No III: The Mortality Test

Winter workers from experimental colonies were caged and divided into five cages (groups). 47 individual workers of group III A received *N. ceranae* spores *p/o*, while 50 workers of group III B received 2 µL of CBPV and 2 µL of *N. ceranae*, with both inoculums being applied *p/o*. 52 workers of group III C received 2 µL of CBPV *p/c*, 44 workers of group III D received 2 µL of CBPV inoculum *p/o* and 42 workers of group III E received 2 µL of macerated CBPV free and *N. ceranae* free bees in RPMI-1640 medium *p/o*, used as negative control bees.

### 3.5. Individual Bee Sample Preparations

Individual bees (n = 558) were placed in separate tubes MagNA Lyser Green Beads (Roche, Mannheim, Germany) and 1 mL of RPMI-1640 (Life Technologies Corporation, Carlsbad, CA, USA) was added to each bee. Each tube was then placed into the MagNA Lyser Instrument (Roche, Mannheim, Germany) and centrifuged at 6,400 rpm for 80 seconds to disrupt bee tissues into suspension. The tubes were transferred onto ice for 1 min and centrifuged second time at 6,400 rpm for 1 min 200 µL of supernatant obtained from the tissue suspension was used for *Nosema* spore counts in the Bürker haemocytometer chamber using phase contrast light microscopy. The remaining bee suspension was centrifuged for 15 min at 2,500 rpm and 140 µL of supernatant was used for extraction of total RNA according to the manufacturer’s protocol (QIAamp viral RNA mini kit, Qiagen, Hilden, Germany). 

### 3.6. CBPV Detection Using One-Step Reverse Transcription and TaqMan Quantitative Real-Time PCR (RT-qPCR)

The primers and probes for the RT-qPCR were previously described by Blanchard *et al.* [13,30]. The forward primer sequence was 5'-CGCAAGTACGCCTTGATAAAGAAC-3' and the reverse primer sequence was 5'-ACTACTAGAAACTCGTCGCTTCG-3'. The TaqMan probe sequence (5'-TCAAGAACGAGACCACCGCCAAGTTC-3') was labeled with the fluorescent reporter dye FAM (6-carboxyfluorescein) at the 5' end and with the fluorescent quencher dye TAMRA at the 3' end. Amplification was carried out in a single step from isolated RNA with Superscript℘ III Platinum^®^ One-Step RT-PCR kit with ROX (Invitrogen, Carlsbad, CA, USA) according to the manufacturer’s instructions. The protocol was optimized using a series of ten-fold dilutions of RNA, varying the volume of RNA added to the final reaction volume, and testing different annealing temperatures as well as the number of PCR cycles used. The determined thermal profile with the highest efficiency of the standard curve was used as follows: the RNA volume of the examined sample in the assay was 5 μL, primer concentration was 800 nM, and the probe concentration was 100 nM, with the final volume of the reaction mix 25 µL. The RT-qPCR was performed on Mx3005P (Stratagene, CA, USA), and the program included a reverse transcription step at 50 °C for 15 min, followed by a denaturation step at 95 °C for 2 min and 45 cycles of 95 °C for 15 s and 55 °C for 1 min. 

### 3.7. Sequence Analysis of CBPV from Different Experimental Groups

Seven randomly selected CBPV positive samples, each from seven different groups (I A, I B, II A, II B, III B, III C, III D) were amplified by conventional RT-PCR as previously described [19] and PCR products were directly sequenced to confirm the identity of the CBPV strain. Briefly, 570 nucleotides long RT-PCR fragments were amplified from extracted RNA using one step RT-PCR kit (Qiagen, Hilden, Germany) and CBPV-specific primers [13]. PCR fragments were then separated using 1.8% gel electrophoresis, excised from the gel and sent for direct sequencing (Macrogen, The Netherlands). Multiple nucleotide sequence alignment was performed using the Clustal W method of the sequence analysis software Lasergene^®^ (DNASTAR, Madison, WI, USA).

### 3.8. Statistics

The Ct value of inoculum and all Ct values of the experimental samples were averaged and evaluated. We used ANOVA single factor analysis to test whether or not the treatments affected the bee mortality. When necessary, this was followed by a Tukey test for pair wise comparisons. This method was applied to the mean values from the treatments in all our experiments. 

## 4. Conclusions

The results of this study revealed replication of virus after inoculation of experimentally caged bees with field CBPV inoculum. The replication in individual bees after *p/o* inoculation was most successful, while the replication of CBPV was less effective when bees were inoculated *p/c.* Dead bees harbored about 1,000 times higher copy numbers of the virus than live bees. Co-infection of workers with CBPV and *N. ceranae* showed increased replication ability for CBPV when using *p/c* inoculation, suggesting a synergistic effect of *N. ceranae* on CBPV replication. The highest level of bee mortality was observed in the group of bees inoculated with CBPV *p/o*, confirming that this virus is an important pathogen responsible for adult bee mortality. In the group of workers simultaneously inoculated with CBPV and *N. ceranae p/o,* we observed the same rate of mortality as in group inoculated with CBPV *p/c*, followed by the group inoculated only with *N. ceranae p/o.* CBPV is widespread and the results of this experimental study confirm that CBPV alone and combined with *N. ceranae,* is an important pathogen.
